# Supplementary material for: Chemoselective Characterization of New Extracellular Matrix Deposition in Bioengineered Tumor Tissues
Source: bioRxiv. 2025 Mar 19:2025.03.18.643336. Preprint. [Version 1] doi: 10.1101/2025.03.18.643336 (PMC11956949; doi:10.1101/2025.03.18.643336)
Supplement: Supplement 1 — Supplementary figure 1. Western blot detection of azido→biotin signal in cellular fractions of dECM-tumors. Supplementary figure 2. Western blot detection of azido→biotin signal in cellular fractions of tumoroids. Supplementary figure 3. Bar graph of human or rat protein intensities in eluate versus input from the dECM-tumors. Supplementary figure 4. An individual-protein-intensity Proteomap generated with all eluate proteins from the dECM-tumor receiving Ac4GalNAz. Supplementary figure 5. Scatter plots of normalized protein intensities in eluate samples from dECM-tumors or tumoroids. Supplementary figure 6. SYPRO Ruby dot blot of total proteins from each sample analyzed in Figure 6D. Supplementary figure 7. Protein-protein physical interaction network functional enrichment analysis with the STRING database. Supplementary figure 8. Bar graphs showing the normalized, imputed protein intensities of (A) PROX-1, (B) PCOLCE2 and (C) TIMP-1 in eluate samples from dECM-tumors (left, red, n=4) and tumoroids (right, blue, n=5). Table 1. Functional annotation clustering of proteins with top 100 abundance from dECM-tumor newsECM. Table 2. Individual protein intensities before or/and after normalization in all three searches. [file media-1.pdf]

Chemoselective Characterization of Biomaterial-regulated New Extracellular Matrix Deposition in Bioengineered Tumor Tissues

Zihan Ling[1,*], Burke Niego[2,*], Qingyang Li[1], Vanessa Serna Villa[1], Dhruv Bhattaram[1], Michael Hu[1], Zhuowei Gong[1], Lloyd M. Smith[2], Brian L. Frey[2,#], and Xi Ren[1,#]

[1] Department of Biomedical Engineering, Carnegie Mellon University, Pittsburgh, Pennsylvania, United States

[2] Department of Chemistry, University of Wisconsin, Madison, Wisconsin, United States

[*] These authors contribute equally to this study.

[#] Correspondence: Xi Ren (xiren@cmu.edu); Brian L. Frey (bfrey@chem.wisc.edu).

Supplementary Data

Supplementary figure 1. Western blot detection of azido→biotin signal in cellular fractions of dECM-tumors.

Supplementary figure 2. Western blot detection of azido→biotin signal in cellular fractions of tumoroids.

Supplementary figure 3. Bar graph of human or rat protein intensities in eluate versus input from the dECM-tumors.

Supplementary figure 4. An individual-protein-intensity Proteomap generated with all eluate proteins from the dECM-tumor receiving Ac_4GalNAz.

Supplementary figure 5. Scatter plots of normalized protein intensities in eluate samples from dECM-tumors or tumoroids.

Supplementary figure 6. SYPRO Ruby dot blot of total proteins from each sample analyzed in Figure 6D.

Supplementary figure 7. Protein-protein physical interaction network functional enrichment analysis with the STRING database.

Supplementary figure 8. Bar graphs showing the normalized, imputed protein intensities of (A) PROX-1, (B) PCOLCE2 and (C) TIMP-1 in eluate samples from dECM-tumors (left, red, n=4) and tumoroids (right, blue, n=5).

Table 1. Functional annotation clustering of proteins with top 100 abundance from dECM-tumor newsECM.

Table 2. Individual protein intensities before or/and after normalization in all three searches. (Separate excel file)

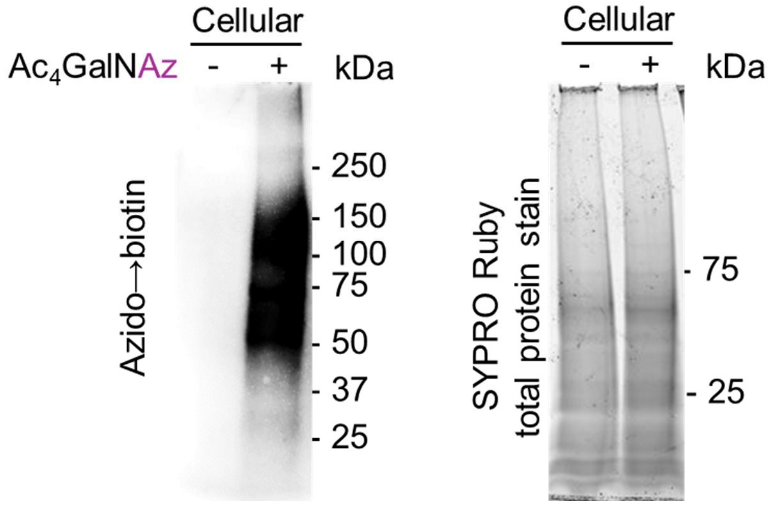

Supplementary figure 1. Western blot detection of azido→biotin signal in cellular fractions of dECM-tumors. Western blot detection of azido→biotin signal in the cellular fractions of dECM-tumors using streptavidin-HRP (left) and SYPRO Ruby staining of total proteins (right).

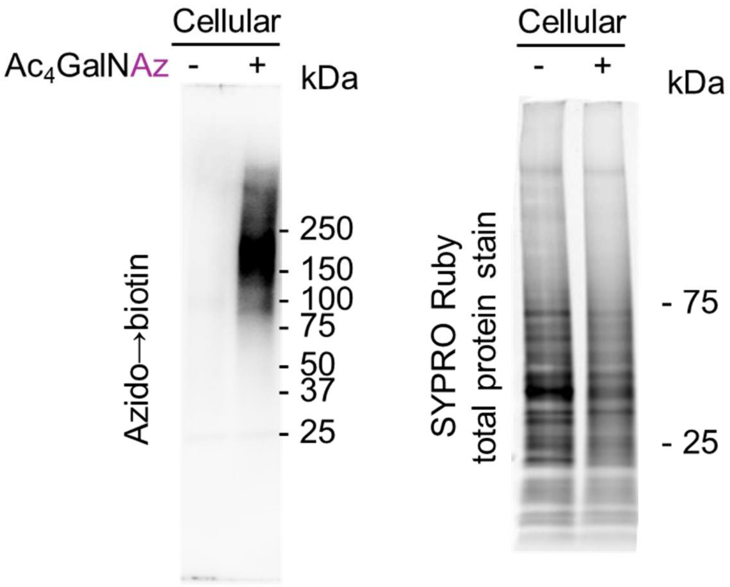

Supplementary figure 2. Western blot detection of azido→biotin signal in cellular fractions of tumoroids. Western blot detection of azido→biotin signal in the cellular fractions of tumoroids using streptavidin-HRP (left) and SYPRO Ruby staining of total proteins (right).

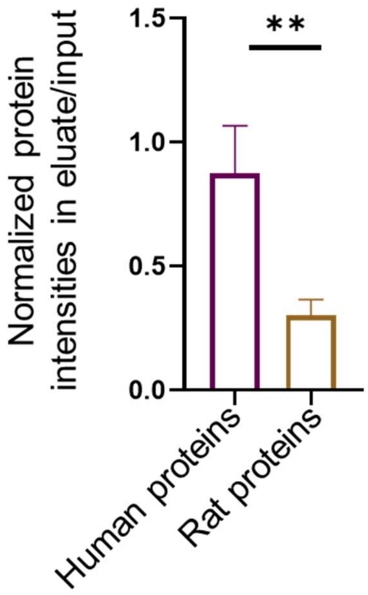

Supplementary figure 3. Bar graph of human or rat protein intensities in eluate versus input from the dECM-tumors. *n*=4. ** $p<0.01$. Data are presented as means ± SD.

Individual protein plot of
all proteins in dECM-tumor eluates

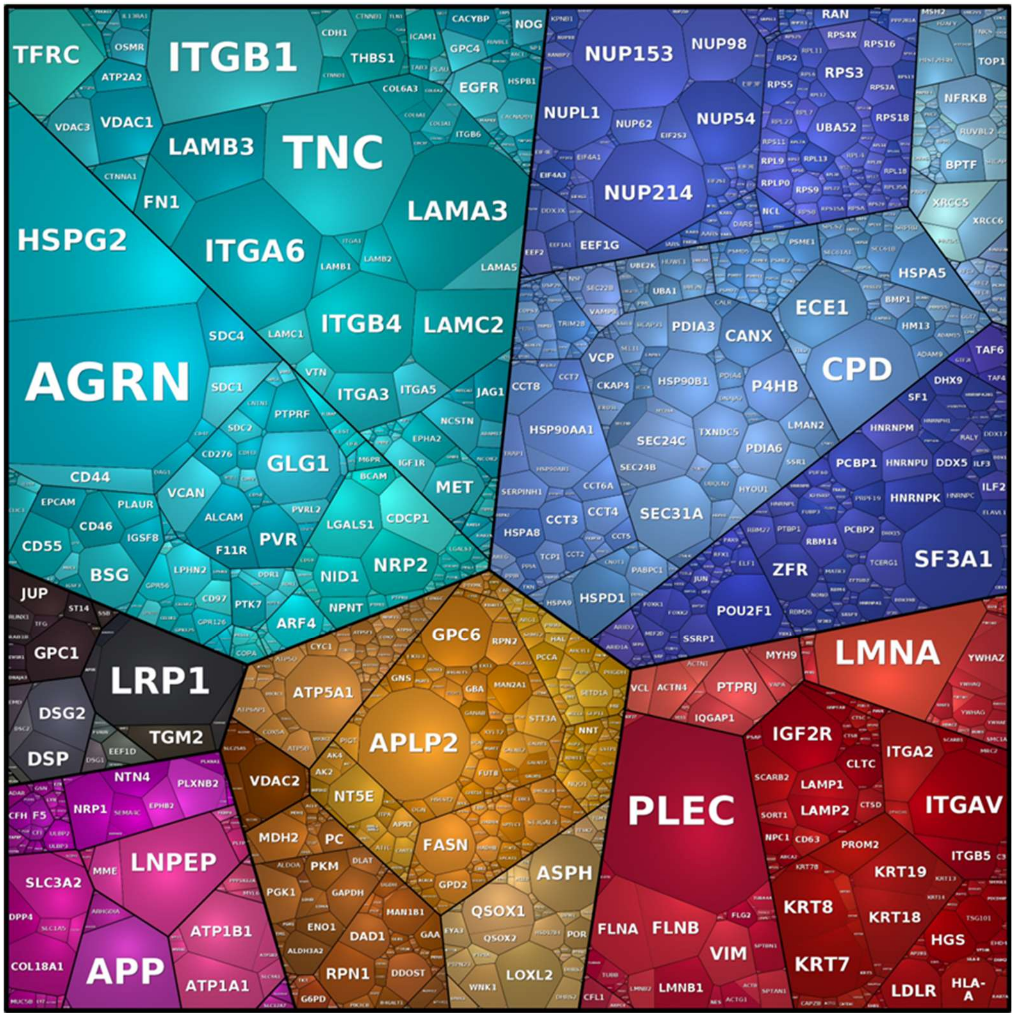

Supplementary figure 4. An individual-protein-intensity Proteomap generated with all eluate proteins from the dECM-tumor receiving $Ac_4GalNAz$. The area of each protein represents its intensity level and color-coded for different proteins.

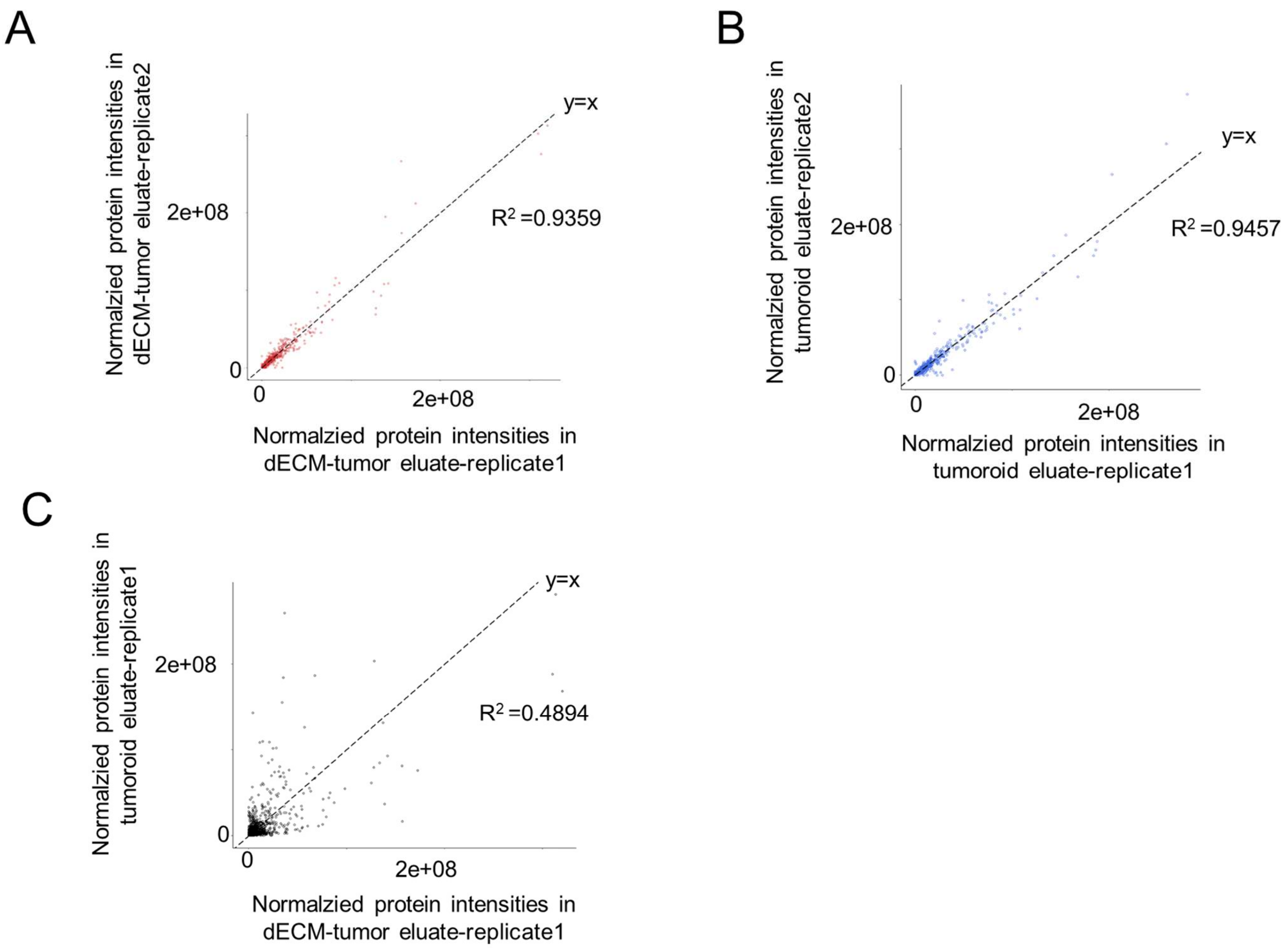

Supplementary figure 5. Scatter plots of normalized protein intensities in eluate samples from dECM-tumors or tumoroids. Scatter plots of protein intensities between (A) two eluate samples from dECM-tumor, (B) two eluate samples from tumoroids, and (C) one eluate sample from dECM-tumor and one eluate sample from tumoroid.

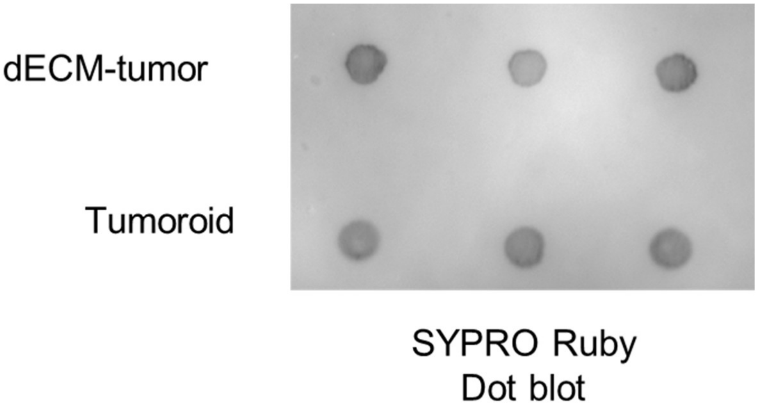

Supplementary figure 6. SYPRO Ruby dot blot of total proteins from each sample analyzed in Figure 6D.

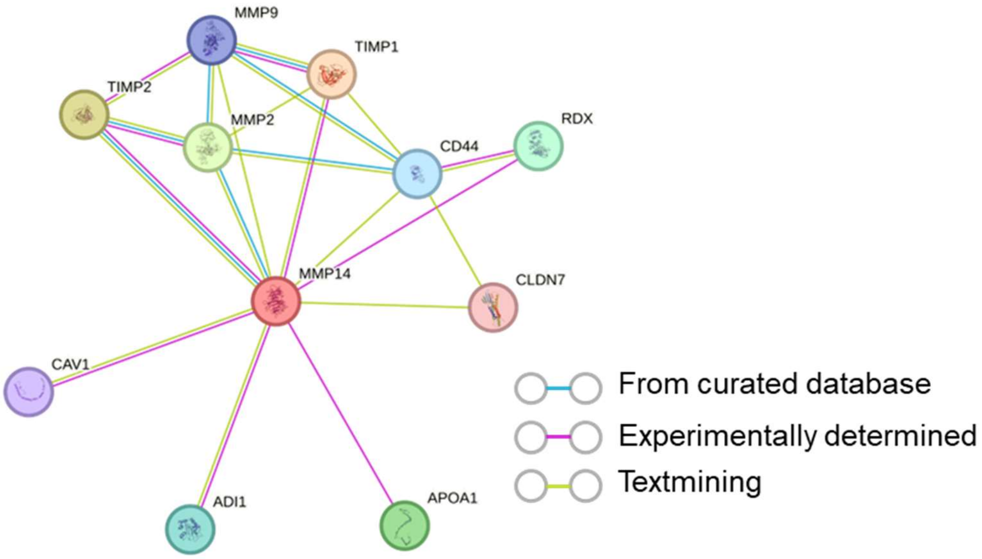

Supplementary figure 7. Protein-protein physical interaction network functional enrichment analysis with the STRING database. The colors of the lines linking two proteins represent the information sources of known or predicted physical interactions.

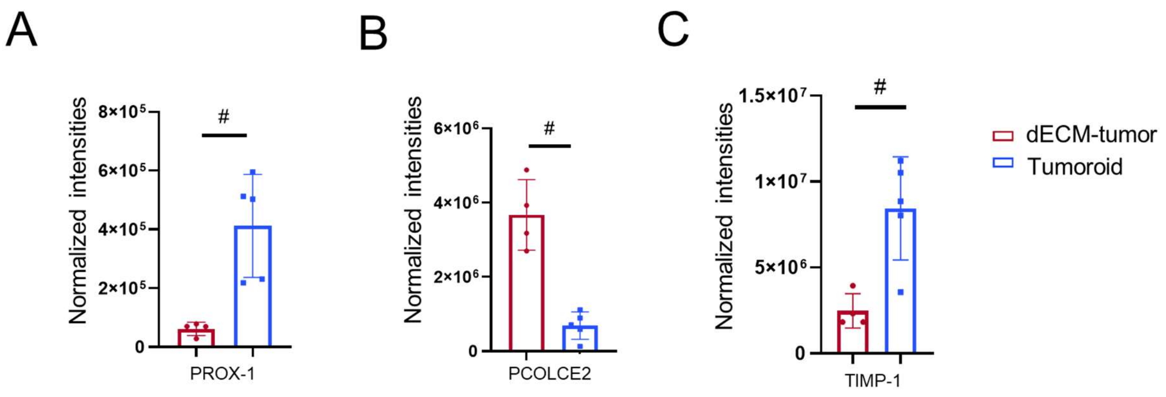

Supplementary figure 8. Bar graphs showing the normalized, imputed protein intensities of (A) PROX-1, (B) PCOLCE2 and (C) TIMP-1 in eluate samples from dECM-tumors (left, red, *n*=4) and tumoroids (right, blue, *n*=5). # q<0.05. Data are presented as means ± SD. Zero values (when the protein was not observed by MS proteomics) were replaced with imputed values as described in Methods (Proteomic Data Analysis).

Table 1. Functional annotation clustering of proteins with top 100 abundance from dECM-tumor newsECM.

GO category	GO term	Count	*p* value	Adjusted *p* value[1]
Annotation Cluster 1 (Enrichment score: 12.41)				
KEGG_PATHWAY	ECM-receptor interaction	18	1.90E-19	2.60E-17
GOTERM_BP_DIRECT	Cell migration	17	4.40E-13	2.50E-10
KEGG_PATHWAY	Proteoglycans in cancer	12	6.70E-07	1.50E-05
Annotation Cluster 2 (Enrichment score: 10.48)				
GOTERM_CC_DIRECT	Collagen-containing extracellular matrix	21	6.40E-15	5.40E-13
GOTERM_CC_DIRECT	Basement membrane	10	1.00E-09	3.10E-08
UP_KW_CELLULAR_ COMPONENT	Extracellular matrix	14	5.70E-09	6.00E-08
Annotation Cluster 3 (Enrichment score: 9.61)				
UP_SEQ_FEATURE	CARBOHYD:N-linked (GlcNAc...) asparagine	57	9.10E-14	1.20E-10
UP_KW_DOMAIN	Signal	59	2.50E-10	5.40E-09
UP_KW_PTM	Disulfide bond	52	6.70E-07	4.50E-06
Annotation Cluster 4 (Enrichment Score: 8.99)				
UP_KW_PTM	Proteoglycan	14	1.70E-13	2.30E-12
UP_KW_PTM	Heparan sulfate	7	5.40E-09	4.90E-08
GOTERM_CC_DIRECT	lysosomal lumen	9	2.50E-08	5.90E-07
GOTERM_CC_DIRECT	Golgi lumen	9	5.00E-08	1.00E-06

[1] *p* values were adjusted by Benjamini correction with FDR<0.05.

Table 2. Individual protein intensities before or/and after normalization in all three searches. "*Search 1* original": original 17 eluates (1 outliner removed) files that underwent GPTMD and were searched using the Human XML database with MBR. "*Search 1* normalization 1": for comparison between Ac_4GalNAz and Vehicle groups, the protein intensities were normalized separately in each treatment group. "*Search 1* normalization 2": proteins from 9 Ac_4GalNAz dECM-tumor and tumoroid eluate samples were normalized all together for their proportional intensities in each group. "*Search 2* original": original 15 dECM-tumor files (inputs and eluates, 1 eluate removed) that underwent GPTMD analysis and were searched using the Human and Rat XML databases without MBR. "*Search 3* original": original 18 dECM-tumor and tumoroid input files that underwent GPTMD analysis and were searched using the Human and Rat XML databases with MBR. "*Search 3* normalization": all input samples were normalized together. The normalization formulas can be found under "Proteomic Data Analysis" in Method section. The table contents are in a separate excel file.
